# PDR Transporter *ABC1* Is Involved in the Innate Azole Resistance of the Human Fungal Pathogen *Fusarium keratoplasticum*

**DOI:** 10.3389/fmicb.2021.673206

**Published:** 2021-06-04

**Authors:** Jasper Elvin James, Erwin Lamping, Jacinta Santhanam, Richard David Cannon

**Affiliations:** ^1^Biomedical Science Programme, Faculty of Health Sciences, Universiti Kebangsaan Malaysia, Kuala Lumpur, Malaysia; ^2^Sir John Walsh Research Institute, Faculty of Dentistry, University of Otago, Dunedin, New Zealand

**Keywords:** azole, PDR transporter, drug efflux, FSSC, *Fusarium keratoplasticum*, fusariosis

## Abstract

*Fusarium keratoplasticum* is arguably the most common *Fusarium solani* species complex (FSSC) species associated with human infections. Invasive fusariosis is a life-threatening fungal infection that is difficult to treat with conventional azole antifungals. Azole drug resistance is often caused by the increased expression of pleiotropic drug resistance (PDR) ATP-binding cassette (ABC) transporters of the ABCG sub-family. Most investigations of *Fusarium* ABC transporters associated with azole antifungal drug resistance are limited to plant pathogens. Through the manual curation of the entire ABCG protein family of four FSSC species including the fully annotated genome of the plant pathogen *Nectria haematococca* we identified PDR transporters *ABC1* and *ABC2* as the efflux pump candidates most likely to be associated with the innate azole resistance phenotype of *Fusarium keratoplasticum*. An initial investigation of the transcriptional response of logarithmic phase *F. keratoplasticum* cells to 16 mg/L voriconazole confirmed strong upregulation (372-fold) of *ABC1* while *ABC2* mRNA levels were unaffected by voriconazole exposure over a 4 h time-period. Overexpression of *F. keratoplasticum ABC1* and *ABC2* in the genetically modified *Saccharomyces cerevisiae* host ADΔΔ caused up to ∼1,024-fold increased resistance to a number of xenobiotics, including azole antifungals. Although *ABC1* and *ABC2* were only moderately (20% and 10%, respectively) expressed compared to the *Candida albicans* multidrug efflux pump *CDR1*, overexpression of *F. keratoplasticum ABC1* caused even higher resistance levels to certain xenobiotics (e.g., rhodamine 6G and nigericin) than *CDR1*. Our investigations suggest an important role for *ABC1* orthologues in the innate azole resistance phenotype of FSSC species.

## Introduction

Species of the *Fusarium solani* species complex (FSSC) are ubiquitous moulds often detected in soil or on plants (Nalim et al., 2011; James et al., 2020) and in indoor plumbing drains (Short et al., 2014). They can infect plants (Coleman et al., 2009), animals (O’Donnell et al., 2016), and humans (Horn et al., 2014). *Fusarium keratoplasticum* and *Fusarium falciforme*, two terrestrial FSSC species, have caused fusariosis of endangered sea turtle eggs (Sarmiento-Ramirez et al., 2014; Gleason et al., 2020). *F. keratoplasticum* and *Fusarium petroliphilum* are the two FSSC species most frequently isolated in the clinic (Herkert et al., 2019; James et al., 2020). FSSC infections are among the most common opportunistic nosocomial mould infections after infections caused by *Aspergilli* (Alastruey-Izquierdo et al., 2013; Lass-Florl and Cuenca-Estrella, 2017). The FSSC accounts for ∼60% of all fusariosis cases worldwide (Guarro, 2013) ranging from superficial localised skin, nail and eye lesions to life-threatening disseminated fungal infections (Dignani and Anaissie, 2004). Invasive fusariosis (IF) usually occurs in immunocompromised patients especially those with hematologic malignancies (Horn et al., 2014). The increasing number of immunocompromised patients (Harpaz et al., 2016) causes heightened concern for invasive mould infections that have high mortality rates (Tortorano et al., 2014; Lass-Florl and Cuenca-Estrella, 2017) and for which treatment options are limited due to the intrinsic triazole antifungal resistance of most FSSC isolates (Gallagher et al., 2003; Espinel-Ingroff et al., 2016; Al-Hatmi et al., 2018).

The triazole voriconazole (VRC) and liposomal amphotericin B either alone or combined are the recommended treatment options for localised infections and IF (Liu et al., 2011; Al-Hatmi et al., 2018). Although most FSSC species are resistant to the majority of azole antifungals (Tupaki-Sreepurna et al., 2017; Herkert et al., 2019), their resistance mechanism(s) remain poorly understood. In *Candida* (Whaley et al., 2016) and *Aspergillus* species (Diaz-Guerra et al., 2003; Wiederhold et al., 2016; Rivero-Menendez et al., 2019), azole resistance is often caused by mutations that lead to the overexpression and/or alteration of the azole antifungal drug target lanosterol 14-α demethylase, *ERG11* (yeasts) or *CYP51* (moulds), an essential enzyme of the ergosterol biosynthesis pathway. The transcription factor AtrR was recently identified to be responsible for the co-regulation of *CYP51A* and the multidrug efflux pump *ABCG1* (also known as *CDR1B*), both of which are major contributors to azole-resistance in *Aspergillus fumigatus* (Hagiwara et al., 2017; Paul et al., 2019). *Aspergilli* have two *CYP51* orthologues (*CYP51A* and *CYP51B*) (Mellado et al., 2001) whereas *Fusarium* spp. have three *CYP51* orthologues (*CYP51A*, *CYP51B* and *CYP51C*) (Becher et al., 2011; Zheng et al., 2019; James et al., 2020). The *CYP51C* orthologue is a unique characteristic of this fungal genus. *CYP51A* and *CYP51B* are sterol 14-α demethylases, but the function of *CYP51C* remains unknown (Fan et al., 2013; Zheng et al., 2019). We have recently reported a 23 bp *CYP51A* promoter deletion that was associated with increased VRC resistance in both clinical and environmental FSSC isolates (James et al., 2020).

Azole resistance is a multifactorial phenomenon. It is mainly determined by the affinity of the azole to the drug target and the expression of multidrug efflux pumps that prevent azoles from reaching their intracellular target (Lamping et al., 2009). High level azole resistance in most pathogenic fungi is caused by the overexpression of efflux pumps belonging to the ATP-binding cassette (ABC) transporter superfamily, the majority of which are pleiotropic drug resistance (PDR) transporters (Cannon et al., 2009; Abou Ammar et al., 2013). In the *Fusarium* species *Gibberella pulicaris* (anamorph: *Fusarium sambunicum*) Abc1 was reported to be a virulence factor that contributed to the tolerance of the phytoalexin rishitin, a defence secondary metabolite, in potato tubers (Fleissner et al., 2002). Orthologues of *Gp*Abc1 appear to play similar roles in many other important *Fusarium* plant pathogens. *F. culmorum* Abc1, for instance, is an important virulence factor (Skov et al., 2004) that protects this plant pathogen against barley phytoalexins and the triazole antifungal, tebuconazole (Hellin et al., 2018). Expression of the Abc1 orthologue, *Fusarium graminearum* Abc3, was dramatically induced by tebuconazole and its deletion caused increased sensitivity to triazole antifungals and reduced virulence towards wheat, maize and barley, which was possibly the result of Abc1 protecting the fungus against yet to be identified phytoalexins (Abou Ammar et al., 2013). The *Gp*Abc1 orthologue Abc1 of the FSSC species *N. haematococca* is also an important virulence factor, the expression of which was dramatically induced by the pea phytoalexin pisatin. But although the deletion of *Nh*Abc1 caused decreased virulence it did not increase sensitivity to any of the 45 antimicrobials tested (Coleman et al., 2011). This prompted the hypothesis that Abc1 orthologues may be important players in the innate azole resistance phenotype of *F. keratoplasticum.*

There are nine eukaryotic ABC protein superfamilies (ABCA to ABCI) according to the Human Genome Organisation (HUGO) nomenclature for ABC proteins (Dean et al., 2001) which has also found wide acceptance in the plant and fungal research community (Verrier et al., 2008; Paumi et al., 2009; Kang et al., 2011). ABCE, ABCF, and “ABCG other” family members are soluble proteins, whereas ABCA, ABCB, ABCC, ABCD, ABCH, and ABCI family members are transporters comprising two homologous halves each with a transmembrane domain (TMD) followed by a nucleotide binding domain (NBD) i.e., (TMD-NBD)_2_. ABCG transporters, however, have an inverted (NBD-TMD)_2_ topology. *Saccharomyces cerevisiae* possesses 30 ABC proteins belonging to six ABC subfamilies: ABCB, ABCC, ABCD, ABCE, ABCF, and ABCG (Paumi et al., 2009). Filamentous fungi typically have twice or three times as many ABC transporters (Kovalchuk and Driessen, 2010; Lamping et al., 2010), but despite their obvious importance, the biological function of most remains unknown.

This study aimed to identify and characterise PDR transporters that may be involved in the azole antifungal drug resistance of the clinically important FSSC species, *F. keratoplasticum*. We used the genome of *Nectria haematococca* mpVI 77-13-4 (Coleman et al., 2009) as the closest relative to search for PDR transporter orthologues most likely associated with azole resistance. During that process we also created a manually curated ABCG protein inventory for four FSSC species and we identified two multidrug efflux pump candidates, *ABC1* and *ABC2*, as the mostly likely candidates responsible for the innate azole resistance phenotype of *F. keratoplasticum*. Using *NhABC1* and *NhABC2* sequences as a guide we isolated *F. keratoplasticum ABC1* and *ABC2*, we studied their expression in response to VRC, and we characterised their efflux pump function in the heterologous host *S. cerevisiae* ADΔΔ.

## Materials and Methods

### Fungal Strains and Culture Conditions

*F. keratoplasticum* 2781, a clinical isolate that is resistant (MIC >32 mg/L) to itraconazole (ITC), posaconazole (PSC) and voriconazole (VRC) (James et al., 2020), was grown on potato dextrose agar (PDA; Oxoid Ltd., Hampshire, United Kingdom) plates incubated at 28°C for 4–7 days. *S. cerevisiae* ADΔΔ (Sagatova et al., 2015) was grown on yeast extract peptone dextrose [YPD; 1% w/v yeast extract (Formedium Ltd., Norfolk, United Kingdom), 2% w/v peptone (Formedium), and 2% w/v glucose (Formedium)] agar plates incubated at 30°C for ∼3 days. *S. cerevisiae* ADΔ/CaCDR1A-GFP overexpressing the prototype fungal multidrug efflux pump *C. albicans CDR1A* with a C-terminal green fluorescent protein (GFP) tag (Lamping et al., 2007) was used as an efflux pump positive control strain. *S. cerevisiae* ADΔΔ uracil prototroph transformants were selected on CSM-URA agar plates containing 2% w/v glucose (Formedium), 0.69% w/v yeast nitrogen base without amino acids (YNB; Formedium), 0.077% w/v yeast complete supplement mixture without uracil (Formedium) and 2% w/v bacto agar (Formedium). Strains used in this study are listed in Table 1.

**TABLE 1:** Fungal strains used in this study.

Strain	Genotype or description	Source
*Fusarium keratoplasticum* 2781	A clinical VRC resistant isolate	James et al., 2020
*S. cerevisiae* AD1-8u^–^	MATα, *PDR1-3, ura3*, *his1*, Δ*yor1*::*hisG*, Δ*snq2*::*hisG*, Δ*pdr10*::*hisG*, Δ*pdr11*::*hisG*, Δ*ycf1*::*hisG*, Δ*pdr3*::*hisG*, Δ*pdr15*::*hisG*, Δ*pdr5*::*hisG*	Decottignies et al., 1998
ADΔ	AD1-8u^–^, Δ*ura3*	Lamping et al., 2007
ADΔ/CaCDR1-GFP	ADΔ, Δ*pdr5*::pABC3-CaCDR1A-GFP	Lamping et al., 2007
ADΔΔ	ADΔ, Δ*his1*	Sagatova et al., 2015
ADΔΔ/FkABC1	ADΔΔ, Δ*pdr5*::pABC3-FkABC1	This study
ADΔΔ/FkABC1-XLmGFPHis	ADΔΔ, Δ*pdr5*::pABC3-FkABC1-XLmGFPHis	This study
ADΔΔ/FkABC2	ADΔΔ, Δ*pdr5*::pABC3-FkABC2	This study
ADΔΔ/FkABC2-XLmGFPHis	ADΔΔ, Δ*pdr5*::pABC3-FkABC2-XLmGFPHis	This study

### ABCG Efflux Pump Candidate Search

ABCG proteins were identified with a BLAST search of the *Nectria haematococca* mpVI 77-13-14 protein database (Coleman et al., 2009) using *S. cerevisiae* S288C Pdr5, Adp1, YOL075C, ModF, and Caf16 and the *C. albicans* ABCG half-transporter orf19.1320 as queries. ABCG transporter topologies were predicted with the Constrained Consensus TOPology (CCTOP) (Dobson et al., 2015) prediction software, and their phylogenetic relationships were determined with Clustal Omega alignments (Sievers et al., 2011) of an ABCG transporter dataset that included representatives of each PDR transporter lineage of Ascomycota (Pezizomycotina and Saccharomycotina) and Basidiomycota species that were extracted from a previous investigation (Lamping et al., 2010). Maximum-likelihood phylogenetic trees with 1000 bootstrap replicates were constructed using a publicly available online tool, the Randomised Axelerated Maximum Likelihood-High Performance Cloud Computing (RAxML-HPC2) (Stamatakis, 2014) available at CIPRES Science Gateway^1^ (Miller et al., 2010).

### Isolation and Characterisation of *FkABC1* and *FkABC2*

Growth of *F. keratoplasticum* 2781 cells and genomic DNA (gDNA) extractions were performed as previously described (James et al., 2020). DNA oligomer primers used in this study are listed in Supplementary Table 1. The Phusion High-Fidelity DNA Polymerase (NEB, MA, United States) was used for PCR amplification of *F. keratoplasticum* gDNA fragments with 45 cycles of denaturation at 98°C for 10 s and a single annealing/extension step at 72°C for 2 min 30 s on a C1000 Touch^TM^ thermal cycler (Bio-Rad, CA, United States).

### Isolation and Characterisation of *FkABC1* and *FkABC2* cDNA ORFs

Logarithmic phase *F. keratoplasticum* cells of a 50 mL potato dextrose broth (PDB) culture incubated at 30°C for 21 h with shaking at 200 rpm were used for total RNA extraction. Removal of traces of gDNA, determination of total RNA concentrations, confirmation of RNA integrity and cDNA synthesis from total RNA extracts were performed as previously described (James et al., 2020). *FkABC1* and *FkABC2* cDNA ORFs were amplified by PCR from cDNA templates using DNA oligomer primers (Supplementary Table 1) that were designed using the gDNA ORF sequences. The PCR amplification conditions were the same as described above. The PCR fragments were analysed by agarose gel electrophoresis and, for DNA sequencing of the PCR fragments, excess oligomer primers were removed by ExoSAP-IT (Applied Biosystems, CA, United States) treatment following the manufacturer’s instructions.

### Quantification of *ABC1* and *ABC2* mRNA Expression Levels in *F. keratoplasticum* Cells Grown in the Presence of VRC

Logarithmic phase *F. keratoplasticum* cells grown in five separate flasks containing 50 mL PDB at 30°C for 21 h with shaking at 200 rpm were incubated with VRC (16 mg/L) for a further 0, 20, 40, 80, and 240 min. Cells were harvested by filtration with a vacuum manifold, the cells were scraped off the filter paper with a spatula, and transferred into a 1.5 mL microcentrifuge tube and immediately snap frozen in liquid nitrogen. Total RNA (1 μg) extracted from these cells was used for first strand cDNA synthesis, and the quantification of mRNA expression levels of individual genes was conducted by real-time qPCR as previously described (James et al., 2020). Oligonucleotide primers used for qPCR amplification of *F. keratoplasticum ABC1, ABC2* and the reference gene *GAPDH* are listed in Supplementary Table 1. The amplification efficiencies of all cDNA amplicons were determined with four 10-fold serial dilutions of first strand cDNA templates (i.e., from 5 to 0.0005 ng of total RNA). They ranged from 100 to 105%. The average amplification cycle value (Cq) of each sample was calculated from two technical replicates. mRNA transcript levels (2^–Δ*Cq*^) were normalised to the reference house-keeping gene *GAPDH*. The fold-change of normalised mRNA expression levels in the presence of VRC relative to logarithmic cells at time zero of VRC induction were calculated using the ΔΔ-Cq method (2^–ΔΔ*Cq*^) (Livak and Schmittgen, 2001).

### Yeast Transformation

*S. cerevisiae* ADΔΔ cells were transformed using a protocol adapted from a previous study (Gietz and Schiestl, 2007). The major improvement for optimised transformation of the ADΔΔ host was the need to reduce the heat-shock temperature from 42 to 30°C. In short, ADΔΔ cells were grown in 250 mL 2 × YPCD (i.e., 2 × YPD plus 0.79 g CSM/L) broth to an optical density (OD_600_) of ∼6, the cells were harvested, washed twice, with 100 and 20 mL, sterile deionised water and slowly resuspended in a 5% (w/v) glycerol, 10% (v/v) dimethyl sulfoxide solution. Competent cells were either kept on ice until further use or stored at −80°C for future transformations. After harvesting 50 μL competent cell aliquots by centrifugation at 17,968 *g* for 2 min the cell pellet was resuspended, by vigorous vortexing for 30 s, with a 360 μL mixture of 296 μL polyethylene glycol/lithium acetate (i.e., 260 μL 50% PEG 3350 plus 36 μL 1 M LiAc), 50 μL salmon sperm carrier DNA (2 mg/mL in 10 mM Tris.Cl, 1 mM EDTA; pH 7.5) and 14 μL of the appropriate DNA fragments (∼0.5–1 μg) and incubated for 60 min at 30°C. The transformed cells were harvested by centrifuging at 17,968 *g* for 10 s, resuspended in 80 μL sterile water, plated onto CSM-URA agar plates, and incubated at 30°C for 2–3 days until uracil prototroph transformants became clearly visible. We routinely obtained 10–100 transformants per μg of the combined DNA fragments.

### Cloning and Heterologous Expression of *FkABC1* and *FkABC2* in *S. cerevisiae* ADΔΔ

**FIGURE 1:**
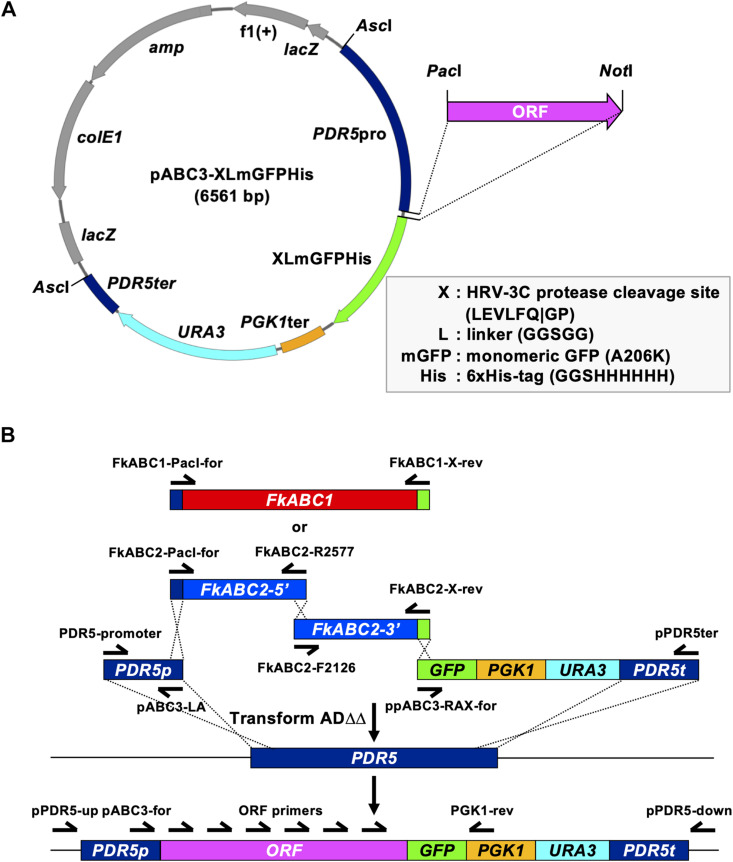
A one-step cloning strategy optimised for the overexpression of fungal PDR transporters in *S. cerevisiae* ADΔΔ. **(A)** The multifunctional pABC3-GFP (Lamping et al., 2007) derivative plasmid pABC3-XLmGFPHis was designed for optimum expression, detection and purification of C-terminally tagged ORFs. Improvements are highlighted in the grey box. pABC3-XLmGFPHis can be used for conventional cloning of any ORF into the *Pac*I/*Not*I restriction sites, as described in Lamping et al., 2007, or it can be used as a template for a much more efficient and faster one-step cloning strategy of any ORF of interest. The grey coloured region represents the pBluescript IISK(+) backbone while the colour-coded region highlights the transformation cassette which comprises the *S. cerevisiae PDR5* promoter followed by the XLmGFPHis double tag, the *PGK1* terminator, the *URA3* selection marker and part of the *PDR5* downstream region. **(B)** *FkABC1* (red) and *FkABC2* (blue) ORFs were PCR amplified from first-strand cDNA using DNA oligomer primers (half arrows) designed to overlap by 25 bp with the left arm (blue) and right arm (green) DNA fragments. The left and right arm fragments were prepared as gel-purified DNA stocks by PCR amplification from plasmid pABC3-XLmGFPHis using the indicated primers. Due to technical difficulties the *FkABC2* ORF was amplified as two separate DNA fragments that overlapped by 25 bp. Equimolar amounts (∼1 μg total) of three (*FkABC1*) or four (*FkABC2*) PCR amplified DNA fragments were used to transform *S. cerevisiae* ADΔΔ and positive Ura^+^ transformants were tested for correct integration of the entire transformation cassette with primers that bound to regions ∼40 bp upstream and downstream of the integration site. Correct integration occurred with 100% accuracy via four (*Fk*ABC1) or five (*Fk*ABC2) homologous recombination events (dashed crossed lines).

A previously designed simple one-step cloning strategy was employed to create *S. cerevisiae* ADΔΔ cells that constitutively overexpressed *FkABC1* or *FkABC2* from single gene copies stably integrated at the genomic *PDR5* locus (Lamping et al., 2017). Briefly, 14 μL mixtures of *FkABC1* or *FkABC2* cDNA ORFs (∼0.5–1 μg), amplified by PCR, and equimolar amounts of the right and left arm DNA fragments, amplified by PCR from pABC3-XLmGFPHis (Figure 1A and Table 2), were combined with 296 μL PEG/lithium acetate and 50 μL salmon sperm carrier DNA to transform ADΔΔ as described above. The ∼1.1 kb “left arm” fragment contained the *S. cerevisiae PDR5* promoter and the “right arm” fragments with (∼2.4 kb) or without (∼1.6 kb) a C-terminal XLmGFPHis double tag contained the *S. cerevisiae PGK1* terminator, the *URA3* selection marker and ∼250 bp of the *PDR5* downstream region (Figure 1B). The overlapping DNA fragments directed correct integration of the entire transformation cassette via three (*FkABC1*) or four (*FkABC2*) homologous recombination events into the genomic *PDR5* locus of *S. cerevisiae* ADΔΔ (Figure 1B). Correct transformants were confirmed by amplifying the 8–9 kb transformation cassettes from 1 μL cell suspensions with primers pPDR5-up and pPDR5-down (Figure 1B) using KOD FX Neo DNA polymerase (TOYOBO Co., Ltd., Osaka, Japan) with 45 amplification cycles. The MICs of fluconazole (FLC) were determined for three independent and correct transformants and if the phenotypes of all three agreed with each other one correct transformant was confirmed by DNA sequencing of the entire ORF (Figure 1B) and selected for all further investigations. The ORFs of *ABC1* and *ABC2* were also cloned as *Pac*I/*Not*I fragments into plasmid pABC3 and pABC3-XLmGFPHis (Table 2), respectively, using traditional cloning protocols (Lamping et al., 2007 and Figure 1A), and stored as plasmid stocks for future applications.

**TABLE 2:** Plasmids used in this study.

Plasmid	Description	Source
pABC3	pBluescript II SK(+)-based plasmid vector	Lamping et al., 2007
pABC3-XLmGFPHis	pABC3 containing a C-terminal mGFPHis double tag	This study
pABC3-FkABC1	pABC3 containing *ABC1* from *F. keratoplasticum* 2781	This study
pABC3-FkABC1-XLmGFPHis	pABC3-XLmGFPHis containing *ABC1* from *F. keratoplasticum* 2781	This study
pABC3-FkABC2	pABC3 containing *ABC2* from *F. keratoplasticum* 2781	This study
pABC3-FkABC2-XLmGFPHis	pABC3-XLmGFPHis containing *ABC2* from *F. keratoplasticum* 2781	This study

### Creation of a Versatile C-Terminal XLmGFPHis Double Tag

An optimised, multifunctional, and versatile pABC3 (Lamping et al., 2007) derivative cloning vector, pABC3-XLmGFPHis (Figure 1A and Table 2), was created for the C-terminal tagging of ORFs with a green-fluorescence/Nickel-affinity double tag. The much improved XLmGFPHis double tag comprises the 8 bp *Not*I restriction enzyme cutting site GCGGCCGC with an extra **G**, introduced by primer design at its 5′ end (**G**GCGGCCGC), to ensure in-frame fusion of the preceding ORF with the C-terminal XLmGFPHis double tag and which is translated as a three amino acid linker (GGR). The GGR linker is followed by an HRV-3C protease cleavage site (TTGGAAGTCTTGTTCCAAGGTCCA = LEVLFQ| GP), a 5 amino acid linker (L = GGTTCTGGAGGCAGT = GSGGS), the monomeric mutant (A206K) version of green-fluorescence protein yEGFP3 (mGFP) (Zacharias et al., 2002; von Stetten et al., 2012), a three amino acid linker (GGTGGCAGT = GGS), and the six-histidine nickel-affinity protein purification tag (CATCATCACCATCATCAT = HHHHHH). This design enables the removal of the mGFPHis double tag to prevent possible interference of the tag in downstream applications. The 5 amino acid linker prevents the possible steric interference of the mGFPHis double tag with the efflux pump function of the attached protein as previously reported for *Candida utilis* Cdr1 (Watanasrisin et al., 2016). The yEGFP3-A206K GFP-variant was created to prevent artificial GFP-dimerisation at high protein concentrations (von Stetten et al., 2012), and the additional 3 amino acid linker between yEGFP3-A206K and the 6His Nickel-affinity tag ensures proper surface exposure of the 6His Nickel-affinity tag to maximise the binding efficiency of the tagged protein to the nickel affinity resin for the possible downstream application of purifying and characterising the structure of the protein of interest.

### Drug Susceptibilities of *S. cerevisiae* ADΔΔ Cells Overexpressing *FkABC1* or *FkABC2*

The following compounds listed from smallest to largest (molecular weights in bracket) were used to determine the substrate specificity of the two possible efflux pumps: anisomycin (ANI; 265), acridine orange (AOR; 265), cycloheximide (CHX; 281), terbinafine (TRB; 291), trichodermin (TRD; 292), FLC (306; Diflucan; Pfizer Laboratories, Auckland, New Zealand), clotrimazole (CLT; 345; Bayer, Osaka, Japan), VRC (349; Cayman Chemical, MI, United States), difenoconazole (DFC; 406), rhodamine 6G (R6G; 479), ketoconazole (KTC; 531) and nigericin (NIG; 725). ANI, AOR, CHX, TRB, TRD, DFC, R6G, KTC, and NIG were purchased from Sigma-Aldrich, MO, United States. To test the drug susceptibilities of cells, 10 mL YPD overnight cultures of yeast cells were diluted 1:20 into 3 mL complete supplement mixture (CSM) pH 7.0 (0.69% YNB, 2% glucose, 0.079% CSM, 10 mM MOPS, 20 mM HEPES; pH 7.0) and grown to mid-logarithmic growth phase (OD_600_ ∼1; ∼10^7^ cells/mL) at 30°C for ∼4 h. Broth microdilution assays of twofold serial dilutions of test compounds in CSM pH 7.0 were used to determine the minimum growth inhibitory concentrations (MIC) of test compounds. The MIC was defined as the lowest concentration of drug that inhibited growth by >90% (Niimi et al., 2004).

### Isolation of Plasma Membranes and Quantification of *FkABC1* and *FkABC2* Expression

*S. cerevisiae* ADΔΔ cells expressing *FkABC1* or *FkABC2* with or without a C-terminal XLmGFPHis double tag were grown in 40 mL YPD liquid medium to mid-logarithmic growth phase (OD_600_ ∼3). A total of ∼40 OD units (1 ODU = 1 mL cell culture of an OD_600_ = 1) of cells were harvested by centrifugation (4,300 *g* for 5 min) at 4°C and the cell pellet was resuspended in 500 μL ice cold homogenising buffer (HB; 50 mM Tris, 1 mM EDTA, 10% glycerol; pH 7.5) freshly supplemented with 1 mM phenylmethylsulfonyl fluoride (PMSF). Samples were kept on ice for 10 min and cells were broken with 1 g ice cold 0.5 mm silica beads using six cycles of vortexing for 1 min followed by 3 min cooling periods on ice. After a 10 min 5,000 *g* centrifugation step to remove cell debris, unbroken cells, nuclei and silica beads ∼450 μL of the supernatant were diluted with an additional 1 mL ice cold HB buffer and the plasma membranes were harvested by centrifugation at 18,000 *g* for 1 h at 4°C and resuspended in 100 μL HB freshly supplemented with 1 mM PMSF. Protein concentrations were determined with the bicinchoninic acid assay (Bio-Rad) using bovine serum albumin as a protein standard. Plasma membrane samples (10 μg each) were separated with SDS-PAGE through an 8% polyacrylamide gel and green fluorescent signals of the GFP-tagged proteins were measured with the Bio-Rad GelDoc system before proteins were visualised by overnight-staining with Coomassie Blue R-250 (Thermo Fisher, MA, United States).

### Confocal Microscopy

Ten microlitres of logarithmic phase ADΔΔ cells overexpressing the C-terminally GFP tagged proteins, grown in 2 mL CSM pH 7.0 at 30°C with shaking at 200 rpm and adjusted to an optical density (OD_600_) of ∼5, were transferred onto a microscope slide that had been coated with a thin agarose film to minimise cell movement during observation. The localisation of the C-terminally GFP tagged proteins was determined by exciting the GFP tag with a 488 nm argon laser (Alexa Fluor 488 channel; 4–7% laser intensities) and detecting the green fluorescence signal with a 517 nm long-pass emission filter using a Zeiss LSM800 confocal laser scanning microscope (Zeiss, Oberkochen, Germany) at a 630 × magnification.

## Results

### *N. haematococca* ABC Protein Inventory and Identification of Putative Multidrug Efflux Pumps, *ABC1* and *ABC2*

Plant and fungal ABCG transporters are commonly known as PDR transporters (van den Brûle and Smart, 2002; Crouzet et al., 2006; Lamping et al., 2010; Balzi and Moye-Rowley, 2019). They transport a large array of compounds across biological membranes, although the biological function of the vast majority of PDR transporters remains to be discovered. The search for PDR transporter homologues in *N. haematococca* mpVI 77-13-14 revealed 21 full-size PDR transporters that could be allocated to five (B, C, F, H1, and H2; Supplementary Figure 1) of the eight previously identified clusters (A–H) of fungal PDR transporters (Cannon et al., 2009; Lamping et al., 2010). The ABCG transporter inventory also included three half-size PDR transporters (Table 3). Alignments of the predicted sequences with the predicted sequences of the entire repertoire of full-size PDR transporters of three closely related FSSC species of the haplotypes FSSC5 (*Fusarium solani*), FSSC10 (*Fusarium* sp.) and FSSC11 (*“Fusarium” solani* f. sp. *pisi*) were used to manually curate the predicted intron/exon boundaries of the JGI curated database sequences. Obvious annotation errors manifested themselves as unusual 20–50 amino acid insertions or deletions in conserved regions of the protein or as an incorrect choice of the ATG start codon that caused short N-terminal truncations of the correct ORF. Mis-annotations (∼40%) of all *N. haematococca*, FSSC5, FSSC10, and FSSC11 ABCG family members, including the soluble “ABCG other” proteins CAF16 and MODF of unknown function, were manually corrected. The corrected sequences are listed in Supplementary File 1. However, most of these sequences have yet to be experimentally verified.

**TABLE 3:** ABCG protein inventory of *Nectria haematococca* mpVI 77-13-14 and their putative function based on their homology to *A. fumigatus* (Af), *S. cerevisiae* S288C (Sc) or *C. albicans* SC5314 (Ca) homologues.

PDR cluster	JGI Protein ID^a^	Predicted topology^b^	Homologue^c^	Localisation of homologue	Function of homologue	References
	Nh95486	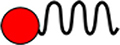	Ca orf19.3120	?	?	Gaur et al., 2005

	Nh90974	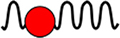	Sc ADP1	Vacuolar	ATP-dependent permease	Purnelle et al., 1991
	Nh123072			membranes and ER		

F	Nh40524	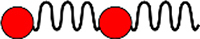	Sc YOL075C/	Part of vacuolar	ATPase-coupled	Decottignies and Goffeau, 1997;
F	Nh68948*		Ca ROA1	membrane and cell periphery	xenobiotic transmembrane transporter	Jiang et al., 2016

B	Nh31707*	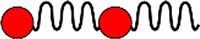	Af AbcG1/AbcC	Plasma membrane	Multidrug transporter	Paul et al., 2017; Esquivel et al., 2020
B	Nh34427* (Abc2)					
B	Nh35467					
B	Nh37125*					
B	Nh48689					
B	Nh56589					
B	Nh63187* (Abc1)					
B	Nh82055					
B	Nh83034					

C	Nh73279	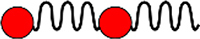	Af AtrI	Plasma membrane	Multidrug transporter	Meneau et al., 2016
C	Nh95029*					
C	Nh103644*					

H1	Nh35868*	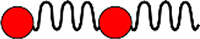	Af AtrF	Part of plasma membrane, cell periphery and mitochondrion	ATPase-coupled xenobiotic transmembrane transporter	Meneau et al., 2016; Esquivel et al., 2020
H1	Nh92267*					
H1	Nh104100					

H2	Nh35089	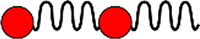	Af AbcH			Esquivel et al., 2020
H2	Nh48703					
H2	Nh54624					
H2	Nh67597*		Af AbcG			

Other	Nh33884	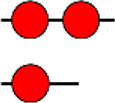	Sc MODF	?	?	Bauer et al., 1999
Other	Nh101582		Sc CAF16			

*^*a*^Asterisks indicate manually corrected sequences.*

*^*b*^Nucleotide binding domains (NBD) are red circles and transmembrane spans (TMS) are shown as curved lines.*

*^*c*^Homologues in A. fumigatus (Af), S. cerevisiae (Sc) and C. albicans (Ca).*

*?Indicate uncategorised/unknown.*

**FIGURE 2:**
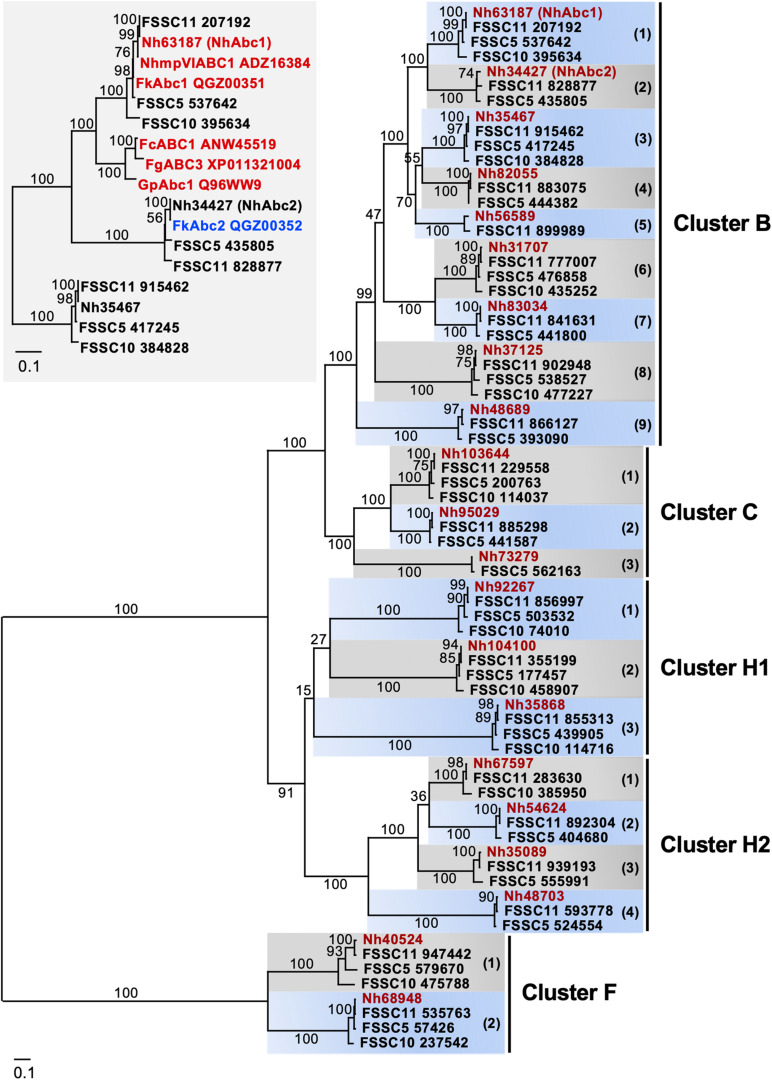
Maximum likelihood phylogram of the entire repertoire of full-size PDR transporters of four FSSC species (i.e., FSSC5, FSSC10, FSSC11, *N. haematococca*). The PDR transporters belong to four (B,C,F,H1,H2) of the eight major clusters (A-H) of fungal PDR transporters (Lamping et al., 2010). The 21 PDR transporters of *Nectria haematococca* (Nh) are highlighted in red. Numbers in brackets on alternating grey or blue background denote nine distinct cluster B, three cluster C, three cluster H1, four cluster H2 and two cluster F lineages, each showing 100% bootstrap support. *Nh*Abc1 and *Nh*Abc2, cluster B lineages 1 and 2, respectively, are shown in brackets. The percentage bootstrap support of 1,000 replicates is shown for all major branches. Inset: The phylogenetic relationship of *Nh*Abc1 and *Nh*Abc2 orthologues of the FSSC with those of related *Fusarium* species that have been shown to be involved in azole antifungal and/or phytoalexin resistance (red). *F. keratoplasticum* (i.e., FSSC2) Abc1 (red) and Abc2 (blue) were also included. Lineage 3 cluster B PDR transporters of the FSSC were used as the outgroup. The percentage bootstrap support of 1,000 replicates is shown for all branches. The scale bars indicate the number of amino acid substitutions per position.

Comparing the entire repertoire of full-size PDR transporters of the four closely related FSSC species (Figure 2 and Table 4) provided interesting insights into PDR transporter evolution in the FSSC. The phylogeny of the concatenated *TEF1-*α and *RPB2* sequences (Supplementary Figure 2) indicated that FSSC10 was the closest living relative of the common ancestor of these four Clade 3 FSSC species. The FSSC2 haplotype *F. keratoplasticum* was closely related to FSSC5 and the FSSC11/*N. haematococca* species pair also separated into a distinct FSSC sub-lineage; both species pairs had relatively good bootstrap support (Supplementary Figure 2). The phylogeny of all full-size PDR transporters of FSSC5, FSSC10, FSSC11, and *N. haematococca* revealed nine distinct cluster B, three cluster C, two cluster F, three cluster H1, and four cluster H2 PDR transporter lineages (Figure 2 and Table 4). The phylogenetic relationship of the individual PDR transporters within the 21 distinct PDR transporter lineages (Figure 2) resembled their species tree (Supplementary Figure 2) suggesting that they are orthologues of possibly similar biological function. *N. haematococca* had all 21 PDR transporter orthologues, FSSC11 had one less cluster C orthologue, FSSC5 had one less cluster B and H2 orthologue, and FSSC10 had the fewest (11) full-size PDR transporters with 4 B, 1 C, 2 F, 3 H1, and 1 H2 cluster orthologues (Figure 2 and Table 4). Table 4 might suggest that the younger FSSC5, FSSC11, and *N. haematococca* species had gained most of the additional PDR transporters in recent evolutionary history. A more careful inspection, however, suggested otherwise. This was particularly clear for cluster C PDR transporters. While all three PDR transporter lineages had 100% bootstrap support (Figure 2) only orthologue 1, one of the two younger branches, was found in all four species but orthologue 3, the ancestor of both cluster C orthologues 1 and 2, was missing in FSSC10 and FSSC11. Thus, FSSC11 had most likely lost orthologue 3 and FSSC10 had most likely lost both cluster C orthologues 2 and 3. Clearly, further analysis is required to carefully ascertain the evolutionary history of PDR transporters of the FSSC.

**TABLE 4:** List of full-size PDR transporters of four FSSC species.

PDR cluster	Orthologue^*a*^	JGI protein ID^*b,c*^			
		
		FSSC10 (11)	FSSC5 (19)	FSSC11 (20)	*N. haematococca* (21)
B	9	−	393090	866127	48689
	8	477227	538527	902948	37125
	7	−	441800	841631	83034
	6	435252	476858	777007	31707
	5	−	−	899989	56589
	4	−	444382	883075	82055
	3	384828	417245	915462	35467
	2	−	435805	828877	34427 **(Abc2)**
	1	395634	537642	207192	63187 **(Abc1)**

C	3	−	562163	−	73279
	2	−	441587	885298	95029
	1	114037	200763	229558	103644

F	2	237542	57426	535763	68948
	1	475788	579670	947442	40524

H1	3	114716	439905	855313	35868
	2	458907	177457	355199	104100
	1	74010	503532	856997	92267

H2	4	−	524554	593778	48703
	3	−	555991	939139	35089
	2	−	404680	892304	54624
	1	385950	−	283630	67597

*Orthologues of individual clusters and species are listed from oldest to youngest from top to bottom and left to right, respectively.*

*^*a*^For the numbering of orthologues refer to Figure 2.*

*^*b*^The protein ID numbers were extracted from the Joint Genome Institute (JGI) database.*

*^*c*^A dash (–) indicates the absence of the indicated orthologue.*

*N. haematococca* mpVI 77-13-14 ORFs Nh63187 (GenBank accession number XP_003048421) and Nh34427 (XP_003044077) were among the closest homologues of *S. cerevisiae* Pdr5, *C. albicans* Cdr1 and *A. fumigatus* AbcG1 (Table 5), but Nh63187 (cluster B orthologue 1) was clearly (100% bootstrap support) the orthologue of *Nh*Abc1, *Fg*Abc3, *Fc*Abc1, and *Gp*Abc1 (inset in Figure 2), all of which are involved in the virulence and/or azole antifungal drug resistance of *N. haematococca* (Coleman et al., 2011), *F. graminearum* (Abou Ammar et al., 2013), *F. culmorum* (Hellin et al., 2018), and *G. pulicaris* (Fleissner et al., 2002), respectively. Thus, the Nh63187 and Nh34427 orthologues were the most likely multidrug efflux pump candidates involved in the innate azole resistance phenotype of *F. keratoplasticum*. To remain consistent with the literature, and to avoid possible confusion, Nh63187 and Nh34427 and their potential *F. keratoplasticum* orthologues were named *ABC1* and *ABC2*, respectively.

**TABLE 5:** *F. keratoplasticum* Abc1 and Abc2 sequence similarities to the indicated Abc1 homologues of other fungi.

		*F. keratoplasticum* 2781^b^			
		
Homologue	UniProt^a^	Abc1		Abc2	
			
		Hom. (%)	Id. (%)	Hom. (%)	Id. (%)
*N. haematococca* mpVI Abc1	F1AWL2	98	98	81	68
*F. culmorum* Abc1	A0A1B1W0H0	88	80	81	68
*F. graminearum* Abc3	I1RL06	88	80	81	68
*G. pulicaris* Abc1	Q96WW9	87	80	80	67
*A. fumigatus* AbcG1^c^	E9RBG1	72	59	73	58
*C. albicans* Cdr1	Q5ANA3	64	48	63	47
*S. cerevisiae* Pdr5	P33302	64	46	65	48

*^*a*^UniProt protein database identifier.*

*^*b*^Hom. indicates homology; Id. indicates identity.*

*^*c*^Also known as AbcC, AbcB, AbcG1, AtrE, Cdr1B.*

### Identification and Characterisation of *F. keratoplasticum ABC1* and *ABC2*

**FIGURE 3:**
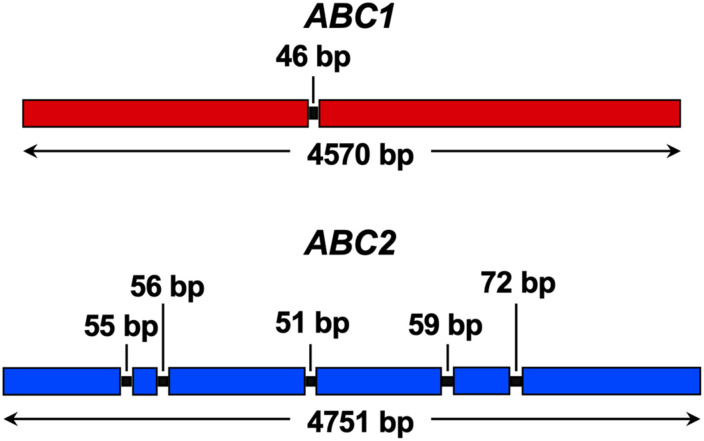
Graphical illustration of *F. keratoplasticum ABC1* (red) and *ABC2* (blue) ORFs. Boxes indicate ORF sequences, black lines indicate introns (intron sizes are listed) and bidirectional arrows indicate the size of the entire ORF including introns.

The *F. keratoplasticum* genome has not been sequenced yet which complicated the amplification and sequencing of the *F. keratoplasticum ABC1* and *ABC2* orthologues. After successful amplification and sequencing gDNA fragments of *F. keratoplasticum ABC1* and *ABC2* with various combinations of forward and reverse primer pairs designed against *N. haematococca ABC1* and *ABC2*, we designed *F. keratoplasticum*-specific primers to amplify and sequence the entire gDNA ORFs including parts of the upstream and downstream sequences. The *F. keratoplasticum* strain Fk2781 *ABC1* (GenBank accession number MN640622) and *ABC2* (MN640623) ORF sequences were 4,570 and 4,751 bp long and their cDNA sequences confirmed the presence of one and five introns, respectively (Figure 3). The number and position of the introns were the same as in *NhABC1* and *NhABC2*. However, careful inspection revealed that the predicted ATG start codons of *N. haematococca ABC1* (Nh63187) and *ABC2* (Nh34427) were possibly incorrect because each had an in-frame ATG start codon further upstream. The eukaryotic translation machinery usually initiates translation at the first AUG start codon the 43S ribosomal preinitiation complex encounters as it scans the gene’s 5′ untranslated region (Hinnebusch, 2011). Thus, the correct ATG start codons for *ABC1* and *ABC2* were quite likely 45 and 123 bp, respectively, further upstream matching the predicted start codons of their orthologues in other FSSC species (Supplementary File 1). This was confirmed by the ability to amplify the *ABC1* and *ABC2* ORFs from cDNA templates with primers that were designed using these corrected ATG start codons. *F. keratoplasticum* Abc1 (1,507 amino acids) and Abc2 (1,485 amino acids) were 98% (Table 5) and 96% identical to their *N. haematococca* counterparts, respectively.

### *FkABC1* mRNA Expression Levels Are Induced by VRC

**FIGURE 4:**
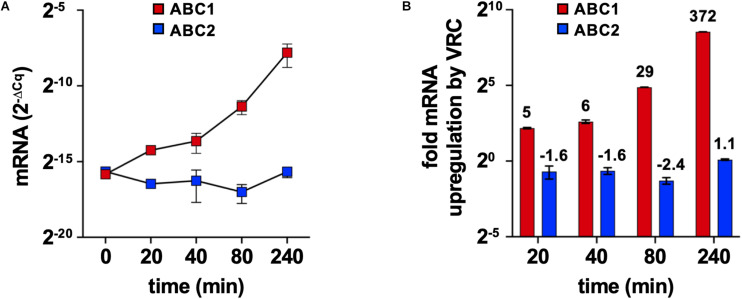
*F. keratoplasticum ABC1* and *ABC2* mRNA expression levels in response to VRC exposure. Logarithmic phase *F. keratoplasticum* 2781 cells were incubated for 4 h at 30°C in PDB medium in the presence of sub-growth inhibitory concentrations of VRC (16 mg/L). **(A)** *GAPDH* normalised *ABC1* and *ABC2* mRNA expression levels (2^–Δ*Cq*^) of *F. keratoplasticum* 2781 cells harvested at the indicated times of VRC induction (16 mg/L). **(B)** Graph of the fold up- or down-regulation of *GAPDH* normalised *ABC1* and *ABC2* mRNA expression levels after 20, 40, 80, and 240 min VRC exposure relative to their mRNA expression levels at time zero. Numbers on the top of each bar indicate the fold changes.

*ABC1* mRNA expression levels in logarithmic phase *F. keratoplasticum* 2781 cells increased exponentially over a 4 h time period in response to high, but sub-growth inhibitory, concentrations (16 mg/L) (James et al., 2020) of VRC, reaching levels that were 372-fold higher after 4 h of VRC induction (Figure 4). Thus, like *S. cerevisiae PDR5*, *C. albicans CDR1* and many other major fungal multidrug efflux pumps *F. keratoplasticum ABC1* mRNA levels appeared to be inducible by a possible multidrug efflux pump substrate. The *ABC2* mRNA expression levels, however, remained low throughout the entire time course and they were not induced by VRC (Figure 4).

### Overexpression and Plasma Membrane Localisation of *Fk*Abc1 and *Fk*Abc2 in *S. cerevisiae* ADΔΔ

**FIGURE 5:**
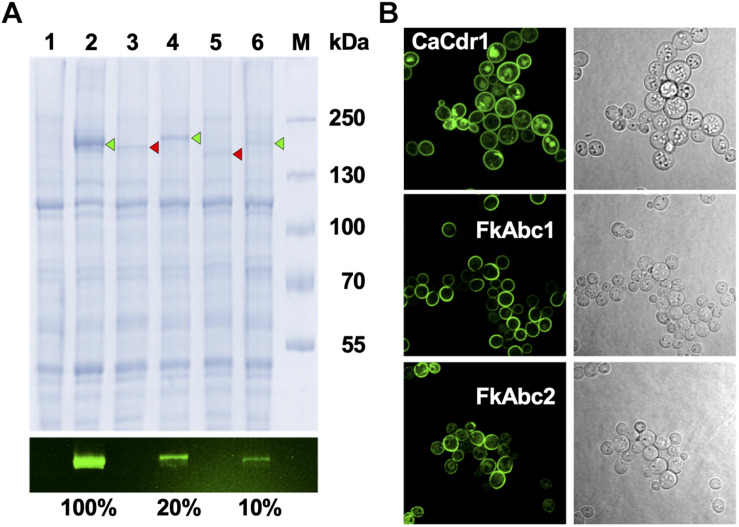
Expression and localisation of *Fk*Abc1 and *Fk*Abc2 in *S. cerevisiae* ADΔΔ. **(A)** SDS-PAGE of plasma membranes (10 μg protein) isolated from *S. cerevisiae* ADΔΔ cells (lane 1; negative control) or from ADΔΔ cells overexpressing *C. albicans* Cdr1-GFP (lane 2; positive control), *F. keratoplasticum* 2781 Abc1 (lane 3), Abc1-XLmGFPHis (lane 4), Abc2 (lane 5), and Abc2-XLmGFPHis (lane 6); lane M is the molecular weight markers [molecular weights (kDa) are indicated]. The image underneath shows the green fluorescence signals of the C-terminally GFP tagged proteins that were used to quantify the expression levels, expressed as % of Cdr1-GFP. The image above was obtained from the same SDS-PAGE gel after Coomassie Blue R-250 staining. Arrowheads point to GFP-tagged (green) or untagged (red) protein bands, respectively. **(B)** Confocal microscopy of ADΔΔ cells overexpressing *Ca*Cdr1-GFP, *Fk*Abc1-XLmGFPHis, and *Fk*Abc2-XLmGFPHis. GFP signals were detected with a LSM800 confocal microscope (Zeiss, Germany) at 630 × magnification. For optimum visualisation the argon laser intensities were adjusted to 4% for *Ca*Cdr1-GFP and 7% for *Fk*Abc1-XLmGFPHis and *Fk*Abc2-XLmGFPHis, respectively. Images on the right are light microscopy images of the same cells.

To investigate the possible efflux pump function of *F. keratoplasticum* Abc1 and Abc2 we expressed the cDNA ORFs in *S. cerevisiae* ADΔΔ, a heterologous host that is highly sensitive to a wide range of xenobiotics and utilises the gain-of-function transcription factor Pdr1-3 mutant allele to constitutively overexpress membrane protein genes integrated at the genomic *PDR5* locus (Lamping et al., 2007). SDS-PAGE of plasma membrane samples and quantification of the fluorescent signals emitted by the C-terminally GFP tagged proteins showed that both Abc1 and Abc2 were expressed at 5–10 times lower levels than *C. albicans* Cdr1 (Figure 5A). To ascertain whether the reduced Abc1 and Abc2 expression levels were possibly caused by incorrect folding and/or plasma membrane localisation we employed confocal microscopy of intact yeast cells (Figure 5B). Both Abc1-GFP and Abc2-GFP localised properly to the plasma membrane (Figure 5).

### Characterisation of the Efflux Pump Activities of *Fk*Abc1 and *Fk*Abc2

**FIGURE 6:**
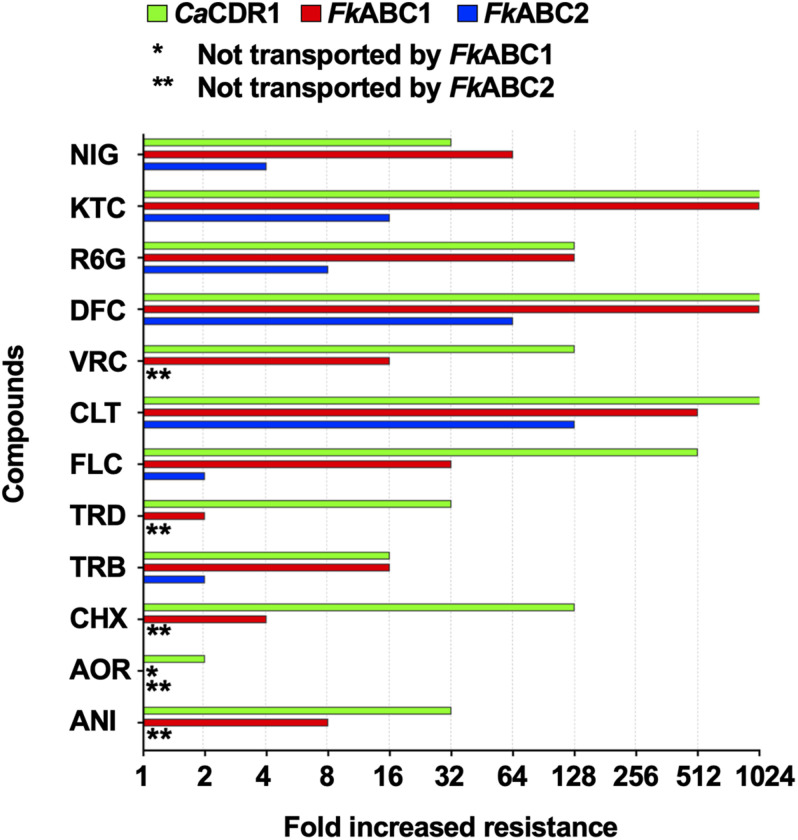
Quantification of drug resistance levels of *S. cerevisiae* ADΔΔ cells overexpressing *CaCDR1* (green), *FkABC1* (red), or *FkABC2* (blue). The increased drug resistance levels (*x*-axis) of 10 test compounds (*y*-axis) were expressed as fold increased MICs relative to the sensitive control strain, *S. cerevisiae* ADΔΔ. The 12 test compounds are arranged, from bottom to top, according to their molecular weight (MW): ANI (MW = 265), AOR (265), CHX (281), TRB (291), TRD (292), FLC (306), CLT (345), VRC (349), DFC (406), R6G (479), KTC (531), and NIG (725). One (*) or two (**) asterisks indicate no transport by *Fk*Abc1 or *Fk*Abc2, respectively. MIC measurements were performed twice and gave identical values.

The FLC susceptibilities of *S. cerevisiae* ADΔΔ cells overexpressing Abc1 and Abc2 with or without the GFP tag were identical, suggesting that the optimised XLmGFPHis double-tag had no detrimental effect on the expression or the efflux pump function of these two PDR transporters. To determine whether Abc1 and Abc2 were indeed multidrug efflux pumps we measured the drug susceptibilities of strains overexpressing Abc1 and Abc2 to 12 xenobiotics. The 12 test compounds varied in size from 265 to 725 Da. They included rather hydrophobic (NIG and TRB) to hydrophilic (CHX or FLC) or charged (R6G; positively charged) molecules and a number of azole antifungals (imidazoles CLT and KTC; triazoles FLC, DFC, and VRC). The MICs for the 12 xenobiotics that target various essential biological processes including translation (CHX), oxidative phosphorylation (R6G), the membrane potential (NIG) and two enzymes of ergosterol biosynthesis (i.e., TRB targets Erg1 and azoles target Erg11) confirmed that both PDR transporters were efficient multidrug efflux pumps (Table 6 and Figure 6). The fold increased drug resistance levels (i.e., the ratio between the MIC of ADΔΔ cells overexpressing the efflux pump and the MIC of the susceptible ADΔΔ host) likely reflect the concentration gradient that the efflux pump helps maintain between the inside and the outside of a cell. It is, therefore, a good proxy for the transportation of a particular compound by the efflux pump. Abc1 overexpression caused 4–1,000-fold increased resistance to all but one (AOR) xenobiotic. Abc1 was particularly efficient in transporting larger compounds (≥306 Da) causing cells to become 64–1,000-fold more resistant to all compounds ≥345 Da apart from VRC (MW 349 Da) the MIC of which was only 16-fold increased (Figure 6 and Table 6). Abc2 overexpression caused significantly lower drug resistance levels for all 12 test compounds, even after accounting for its ∼2-fold lower expression level. However, it too was more efficient in transporting larger compounds. Abc2 overexpression did not increase the MICs for most of the six smallest test compounds (265–306 Da) apart from a twofold increased MIC for TRB (291 Da) and FLC (306 Da). Abc2 expression caused, however, significantly increased MICs for compounds ≥345 Da, again with the exception of VRC (349 Da) which was not transported at all. Abc2 overexpression caused a 64-fold increased MIC for DIF, an eightfold increased MIC for R6G, a 16-fold increased MIC for KTC and a fourfold increased MIC for NIG. For most xenobiotics, *Ca*Cdr1 expression caused significantly higher resistance levels than cells expressing Abc1 or Abc2. But despite the 5-times lower expression level of Abc1 cells demonstrated equally high (TRB, DFC, R6G, KTC) or even slightly higher (NIG; twofold) resistance than cells overexpressing CaCdr1 (Figure 6).

**TABLE 6:** Drug susceptibilities of *S. cerevisiae* ADΔΔ (control) and ADΔΔ cells overexpressing *FkABC1*, *FkABC2*, or *CaCDR1A*.

Compound^a^	MW (Da)	MIC (mg/L)			
		
		ADΔΔ	*FkABC1*	*FkABC2*	*CaCDR1A*
ANI	265	0.5	4	0.5	>16
AOR	265	8	8	8	16
CHX	281	0.031	0.125	0.031	4
TRB	291	4	>64	8	>64
TRD	292	0.25	0.5	0.25	8
FLC	306	1	32	2	>512
CLT	345	0.004	2	0.5	>8
VRC	349	0.031	0.5	0.031	>4
DFC	406	0.001	1	0.063	>1
R6G	479	1	>128	8	128
KTC	531	0.004	4	0.063	8
NIG	725	0.5	32	2	16

**^*a*^Anisomycin (ANI), acridine orange (AOR), cycloheximide (CHX), terbinafine (TRB), trichodermin (TRD), fluconazole (FLC), clotrimazole (CLT), voriconazole (VRC), difenoconazole (DFC), rhodamine 6G (R6G), ketoconazole (KTC), and nigericin (NIG).**

## Discussion

The *N. haematococca* genome contained 26 ABCG proteins (Table 3 and Supplementary File 1), 21 of which were full-size PDR transporters, more than twice the number typically found in Saccharomycotina species (1–9) and also significantly more than in most other Pezizomycotina species (1–20) including *A. fumigatus* which has only 12 full-size PDR transporters (Lamping et al., 2010). All four FSSC species (i.e., FSSC5, FSSC10, FSSC11, and *N. haematococca*) also had *S. cerevisiae CAF16* and *MODF* orthologues, conserved soluble ABCG proteins that lack a transmembrane domain. They also had one “true” half-size ABCG transporter (i.e., *C. albicans* ORF 19.3120 orthologues) and two *S. cerevisiae ADP1* homologues (Table 3 and Supplementary File 1). Among the full-size PDR transporters all four FSSC species had two cluster F and three cluster H1 PDR transporter orthologues. However, the number of cluster B, C and H2 PDR transporters varied significantly between individual species. FSSC10 had 4, 1, and 1, FSSC5 had 8, 3, and 4, FSSC11 had 9, 2, and 4 and *N. haematococca* had 9, 3, and 4 cluster B, C, and H2 PDR transporters, respectively (Table 4 and Supplementary File 1). The conservation of two soluble ABCG protein orthologues (*CAF16*, *MODF*), three “half-size” ABCG transporter orthologues (Ca19.3120, 2 *ADP1*) and five full-size cluster F (2 YOL075C) and H1 (3) PDR transporters suggests an important function for these 10 ABCG proteins that appear to be conserved among all FSSC species. In contrast, the significant variation of cluster B (4, 8, 9, 9), C (1, 3, 2, 3), and H2 (1, 4, 4, 4) PDR transporters in FSSC10, FSSC5, FSSC11, and *N. haematococca*, respectively, suggests that these transporters were gained or lost during the adaptation of individual species to their natural habitat and/or host organism. It is tempting to speculate that some PDR transporters may have been gained or lost and/or modified in response to the excessive use of agricultural fungicides. An indication that this may indeed be the case was recently demonstrated for the possible azole resistance mechanisms in the fungal plant pathogen, *F. culmorum* (Hellin et al., 2018). The authors noted consistently higher (∼3-fold) *FcABC1* expression levels in tebuconazole treated triazole resistant field isolates compared to field isolates that were considered to be triazole susceptible. Investigations into the possible selection of azole resistant *F. keratoplasticum* clinical isolates (James et al., 2020) due to the overuse of azole antifungals by the agricultural sector is certainly warranted. About 40% of the predicted ORF sequences contained mis-annotations (Supplementary File 1) mainly due to inaccurate ATG start codon and intron-exon boundary predictions. Unfortunately, genome mis-annotations are still all too common (Salzberg, 2019). Correct annotations of PDR transporters are further complicated by frequent gene duplication events that lead to tandem arrays of multiple ORFs with often very similar sequences that are difficult to resolve (Watanasrisin et al., 2016; Lamping et al., 2017). This is why whole genome sequencing should be accompanied with RNA-sequencing to obtain full-length transcripts and improve gene annotations (Salzberg, 2019).

The zinc cluster transcription factors Pdr1 (Balzi et al., 1994) and Tac1 (Coste et al., 2004; Liu et al., 2018) are responsible for the upregulation of the prototype fungal PDR transporter genes *S. cerevisiae PDR5* and *C. albicans CDR1*, respectively. Zinc cluster transcription factors are only found in fungi. They typically bind to DNA binding motifs comprising direct or everted CGG repeats (MacPherson et al., 2006). Pdr1 orthologues bind to the pleiotropic drug resistance element (PDRE) TCCGCGGA in *S. cerevisiae* (Katzmann et al., 1994) and TCCACGGA in *C. glabrata* (Paul et al., 2011) and *C. albicans* Tac1 binds to the *Candida* drug resistance element (CDRE) CGGN_4_CGG (de Micheli et al., 2002; Coste et al., 2004). Similar drug response elements are also found in filamentous fungi. In *A. fumigatus*, the transcription factor AtrR is involved in azole resistance of clinical isolates. AtrR binds to CCGN_12_CGG promoter response elements and upregulates, among many other genes, *CYP51A*, *CYP51B*, and the PDR multidrug efflux transporter, *CDR1B/ABCG1* (Hagiwara et al., 2017; Paul et al., 2019). Our experimental evidence suggests that a similar transcription factor may be responsible for the upregulation of *ABC1* in response to VRC in *F. keratoplasticum*.

Previous investigations of PDR transporters involved in virulence and/or azole resistance of various *Fusarium* species included the creation of gene knock-out strains (Coleman et al., 2011; Abou Ammar et al., 2013), the analysis of transcript levels in *F. graminearum* in response to tebuconazole (Becher et al., 2011), and the *in vitro* adaptation of strains to azole exposure (Hellin et al., 2018). These investigations highlighted the importance of *F. keratoplasticum* Abc1 orthologues in virulence and/or azole resistance in related plant pathogens. Gene knock-out investigations are, however, often hampered by the presence of additional PDR transporters with overlapping transport function that can “mask” the function of the PDR transporter under investigation, as noted for *Nh*Abc1 (Coleman et al., 2011). Although gene knock-out of *N. haematococca ABC1* attenuated virulence in garden peas and caused increased sensitivity to the pea phytoalexin pisatin, it did not cause increased sensitivity to any of 45 antimicrobials tested, although it did cause increased sensitivity to the related potato phytoalexin rishitin (Coleman et al., 2011). Overexpression of PDR transporters in the heterologous host *S. cerevisiae* ADΔΔ eliminates any such masking effects because the deletion of seven ABC transporters makes ADΔΔ exquisitely sensitive to xenobiotics. This, together with Pdr1-3 which causes the constitutive overexpression of plasma membrane PDR transporters makes ADΔΔ the optimal host for studying the efflux pump function of fungal PDR transporters (Nakamura et al., 2001; Lamping et al., 2007). We have expressed and studied the efflux pump function of numerous PDR transporters from a variety of fungal pathogens including the Saccharomycotina species *C. albicans*, *C. glabrata*, and *C. krusei* (Lamping et al., 2007; Lamping et al., 2017), the Pezizomycotina species *Penicillium marneffei* (Panapruksachat et al., 2016) and the Basidiomycota *Cryptococcus neoformans* (Lamping et al., 2007).

The successful expression of *F. keratoplasticum* Abc1 and Abc2 in *S. cerevisiae* was quite remarkable. Previous attempts to express the *P. marneffei* multidrug efflux pump Abc1 caused significantly lower expression levels (∼3% of *Ca*Cdr1) and also much lower (4–8-fold) antifungal resistance levels. And previous attempts (Paul and Moye-Rowley, 2013; Esquivel et al., 2020) to express *A. fumigatus* PDR transporters in a different *S. cerevisiae* host using either a high-copy plasmid pYES2 with a galactose inducible promoter (Esquivel et al., 2020) or a low-copy plasmid under the control of a copper-inducible promoter (Paul and Moye-Rowley, 2013) were even less successful. Using the copper-inducible promoter neither *A. fumigatus* AbcA nor AbcB, better known as AbcG1 or Cdr1B (Table 5), could be expressed in that host. Only codon-optimisation combined with an increased growth temperature (37°C) enabled low AbcB expression and ∼2-fold increased FLC resistance levels (∼1–2 mg/L) (Paul and Moye-Rowley, 2013). Recent attempts to study the efflux pump function of six *A. fumigatus* PDR transporters (AbcA, AbcC, AbcF, AbcG, AbcH, and AbcI) in a similar *S. cerevisiae* host (i.e., Δ*PDR5*) were more successful, although the highest resistance levels achieved toward any of the 26 compounds tested were only 16-fold greater than the sensitive host (Esquivel et al., 2020). The expression levels of these genes were not investigated. Although *Fk*Abc1 and *Fk*Abc2 were expressed at levels 5–10-times lower than *Ca*Cdr1 their overexpression in our genetically modified host ADΔΔ caused up to 1,000-fold increased drug resistance levels that were similar to, or in some cases even higher than, those of cells overexpressing *Ca*Cdr1 (Table 6 and Figure 6). The superior performance of our yeast expression system is possibly due to the Pdr1-3 transcription factor that upregulates not only Pdr5 but also a plethora of other genes that ensure that the right amount and types of lipids are produced to accommodate correct folding, trafficking and function of Pdr5 and related PDR transporters in the plasma membrane of *S. cerevisiae*. Further advantages may be the insertion of 16 additional residues between the GFP tag and the protein of interest and the stable integration of a single gene copy into the genome of ADΔΔ. The superior efflux pump activities of Abc1 and the fact that *Fk*Abc1 orthologues *Nh*Abc1, *Fg*Abc3, *Gp*Abc1, and *Fc*Abc1 (Figure 2) are important virulence factors that protect these organisms from phytoalexins and azole antifungals suggest a critically important efflux pump function of *Fk*Abc1 orthologues. But the poor conservation of *Fk*Abc2 orthologues in FSSC species, the inability of *Fk*Abc2 to efflux VRC, and the fact that *FkABC2* mRNA expression levels were not induced by VRC suggest a more refined efflux pump function for *Fk*Abc2.

In summary, we have created a superior membrane protein expression technology in the eukaryotic model organism *S. cerevisiae* that allows the characterisation of fungal PDR transporters in a background devoid of “masking” efflux pumps. The use of this technology revealed that both fungal cluster B PDR transporters, *Fk*Abc1 and *Fk*Abc2, are multidrug efflux pumps. However, *Fk*Abc1 appears to be the major *F. keratoplasticum* multidrug efflux pump that quite possibly protects cells from phytoalexins and, importantly, contributes to the innate azole resistance phenotype of *F. keratoplasticum*. The conservation of *Fk*Abc1 orthologues indicates a similar role in all other *Fusarium* species including species of the FSSC. Future investigations should confirm these observations.

## Data Availability Statement

DNA sequences generated in this study have been made publicly available at GenBank. The datasets for the phylogenetic analysis will be made available upon request, without undue reservation, to any qualified researcher.

## Author Contributions

JJ and EL performed the experiments, analysed the data, and wrote the manuscript. JS and RC provided overall guidance and edited the manuscript. All authors reviewed and approve the final manuscript.

## Conflict of Interest

The authors declare that the research was conducted in the absence of any commercial or financial relationships that could be construed as a potential conflict of interest.

## Footnotes

1 www.phylo.org
