# Supplementary material for: PDR Transporter ABC1 Is Involved in the Innate Azole Resistance of the Human Fungal Pathogen Fusarium keratoplasticum
Source: Front Microbiol. 2021 Jun 4;12:673206. doi: 10.3389/fmicb.2021.673206 (PMC8211738; doi:10.3389/fmicb.2021.673206)
Supplement: Supplementary file 1 [file Data_Sheet_1.docx]

**Supplementary Table S1.** DNA oligomer primers used in this investigation.

| **Name**^a^ | **Sequence (5’ to 3’)^b^** |
| --- | --- |
| *F. keratoplasticum gDNA ORF amplification and DNA sequencing* | |
| NhABC1-(–F211) | AGCCTCGTTGTGGTCAGTGTCAATTTGGAC |
| NhABC1-R270 | AGAGCAGCACCATCACCCTGAAAGTTCTGC |
| NhABC1-F1182 | TTACGACCTGTTCGACAAGGCCCTG |
| NhABC1-R1285 | GCGCAGTCATCGAGGTCAGGAAATC |
| NhABC1-F2422 | GCGCAACGTCGGAATCATCATCGCT |
| NhABC1-R2507 | AGACAGCAGGCATGTTTCCACGTCG |
| NhABC1-F3650 | GCCTTTGAGCAGTACTGGCGAACTC |
| NhABC1-R3730 | CTGGTTCTGCAAGCCCTGCATGGTC |
| NhABC1-F4295 | AGGTCACATGCGCAGCCAACGAATACGTCG |
| NhABC1-(+R277) | TCAGTTCATGCTGTCGTCATGGCCTTACTG |
| NhABC2-(–F81) | GTACCACAACAGTCGGCAGTTAACCATTGC |
| NhABC2-F65 | GCAACTTTCCTCTACCGGGAACTGC |
| NhABC2-R497 | GACCGTCGAATTGTCGAAGAATGTCAATGC |
| NhABC2-F1170 | GCAGATCTACTTTGGCCCAGCATCC |
| NhABC2-R1279 | CGGGTGTTCGTGGTGGATTACATTC |
| NhABC2-F2359 | GCCACCGAGTATATCACGGCCAAGA |
| NhABC2-R2461 | TGTTGTTGTCGGGCTTTCTCACGAC |
| NhABC2-F3463 | GCTCACGACCCTGCCTCTTATATCG |
| NhABC2-R3555 | GCTGACGGAGATGCAGAGGATGAAC |
| NhABC2-F4223 | TGCGAACCAGACTTGTGGCGACTATATGGC |
| NhABC2-(+R202) | AGTTGCGATTTCATACCTTCCCAGCCAAGC |
| *F. keratoplasticum cDNA ORF amplification and DNA sequencing* | |
| FkABC1-PacI-for | CGCTCGTTCGAAAGACTTAATTAAAAAATGCCTGCCTCCCCAAACCCCAGCC |
| FkABC1-F573 | GCGACGGATCGACATTCTTCGAGGC |
| FkABC1-F1309 | GATTTCTTGACCTCGATGACTACGC |
| FkABC1-F2101 | TTCGTCATTCCCGTCGATTACATGC |
| FkABC1-F2802 | AATGCTTGTCGATGGCAAGATTCGC |
| FkABC1-F3521 | CTGCCCATGACAACGACCACGGTGC |
| FkABC1-F3561* | CCTTCTGGGAGCAGCTTAAA |
| FkABC1-R3631* | TGAAGAGAGCAACCGAAATACA |
| ppABC3-FkABC1-X-rev | CCTTGGAACAAGACTTCCAAGCGGCCGCCTGACTGCTTCTGCTTCTTCCCCTTG |
| FkABC1-NotI-rev | CCAATAATTCCAAAGAAGCGGCCGCATTTATGACTGCTTCTGCTTCTTCCCCTTG |
| FkABC2-PacI-for | CGCTCGTTCGAAAGACTTAATTAAAAAATGATGCCGAAACCATTGGAAAAAC |
| FkABC2-F689 | TACCGGCTGACGAGATGCATACTGC |
| FkABC2-F1384 | GAGATCGAAGAATACAAGACGCAGC |
| FkABC2-F2126 | TCATGGTCAACGAATTCCACAACCG |
| FkABC2-R2577 | GACTTTGACGTCGTAGCAGACGCTG |
| FkABC2-F2846 | TGCATCTCGAGACCAGTACTGTTCG |
| FkABC2-F3549 | AGACCAATTCTTCGTCGTCATTCGC |
| FkABC2-F4100* | ATTGGATCTCGGCTGTCTTATC |
| FkABC2-R4166* | GTAGTCGCTACAAGTCTGGTTC |
| ppABC3-FkABC2-X-rev | CCTTGGAACAAGACTTCCAAGCGGCCGCCGAGCTTCTTATTTCTCTTGGGCATT |
| FkABC2-NotI-rev | CCAATAATTCCAAAGAAGCGGCCGCATTTAGAGCTTCTTATTTCTCTTGGGCATT |
| FkGPD1-F829* | CGAGAAGGGTGCTTCTTACGACGAG |
| FkGPD1-R1050* | AGTAACCCCACTCGTTGTCGTACCA |
| *One-step cloning strategy of ORFs into S. cerevisiae AD∆∆* | |
| pPDR5-up | GCATAAAACAGAGAGGCGATATAGG |
| PDR5-promoter | CACACACATATATATAAGCCTGAATGC |
| pABC3-for | ATAAATTGGCAACTAGGAACTTTCG |
| pABC3-LA | TTTTTAATTAAGTCTTTCGAACGAGCG |
| ppABC3-RAX-for | GCGGCCGCTTGGAAGTCTTGTTCCAAGG |
| PGK1-rev | TTTCGGATAAGAAAGCAACACCTGG |
| pPDR5ter | TTTAGGCACTCTTGCTAACCAGTAGA |
| pPDR5-down | AGAAGACGGTTCGCCATTCGGACAG |

^a^ Numbers indicate the ORF position that the 3’ end of the indicated primer binds to; primers binding at the indicated positions upstream or downstream of an ORF are marked with a minus (upstream) or plus sign (downstream) and placed in brackets.

^b^ Underlined sequences in black are part of *S. cerevisiae PDR5* genomic sequences, in red are the cloning sites.

* Primers used for qPCR.


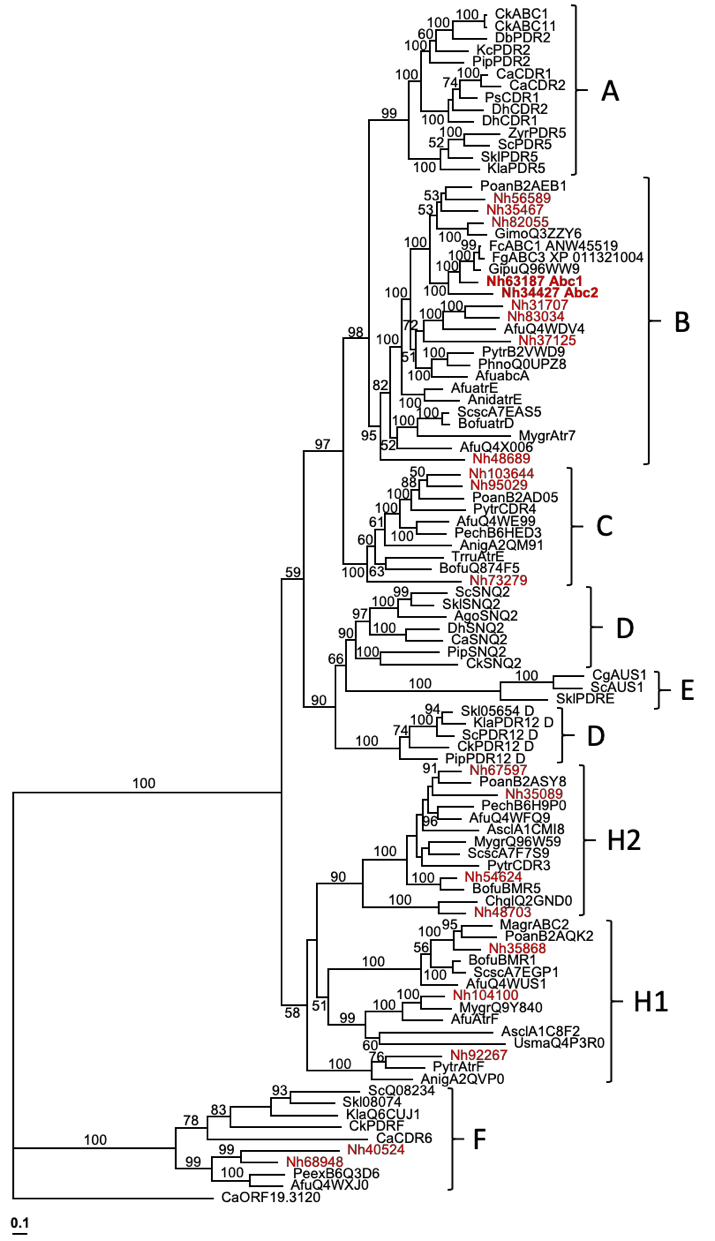


**Species abbreviation:**

Afu *Aspergillus fumigatus*

Ago *Ashbya gossypii*

Anid *Aspergilus nidulans*

Anig *Aspergillus niger*

Ascl *Aspergillus clavatus*

Bofu *Botryotinia fuckeliana*

Ca *Candida albicans*

Cg *Candida glabrata*

Chgl *Chaetomium globosum*

Ck *Candida krusei*

Dh *Debaryomyces hansenii*

Fc *Fusarium culmorum*

Fg *Fusarium graminearum*

Gipu *Gibberella pulicaris*

Kc *Kuraishia capsulata*

Kla *Kluyveromyces lactis*

Magr *Magnaporthe grisea*

Mygr *Mycosphaerella graminicola*

Nh *Nectria haematococca*

Pech *Penicillium chrysogenum*

Peex *Penicillium expansum*

Phno *Phaeosphaeria nodorum*

Pip *Pichia pastoris*

Poan *Podospora anserina*

Ps *Pichia stipites*

Pytr *Pyrenophora tritici-repentis*

Sc *Saccharomyces cerevisiae*

Scsc *Sclerotinia sclerotiorum*

Skl *Saccharomyces kluyveri*

Trru *Trichophyton rubrum*

Usma *Ustilago maydis*

**Supplementary Figure S1.** Maximum likelihood phylogram of representative fungal PDR transporters of clusters A, B, C, D, E, F, H1, and H2, selected from Lamping et al., (2010). Nomenclature: initial letters are abbreviations of the species name followed by their unique protein identifier extracted from either Uniprot or GenBank. *Nectria haematococca* mpVI 77-13-14 PDR transporters are highlighted in red with Abc1 and Abc2 in bold. The percentage branch support of 1,000 bootstrap replicates is only shown for those branches that are >50% supported. Scale bar indicates the number of amino acid substitutions per position.


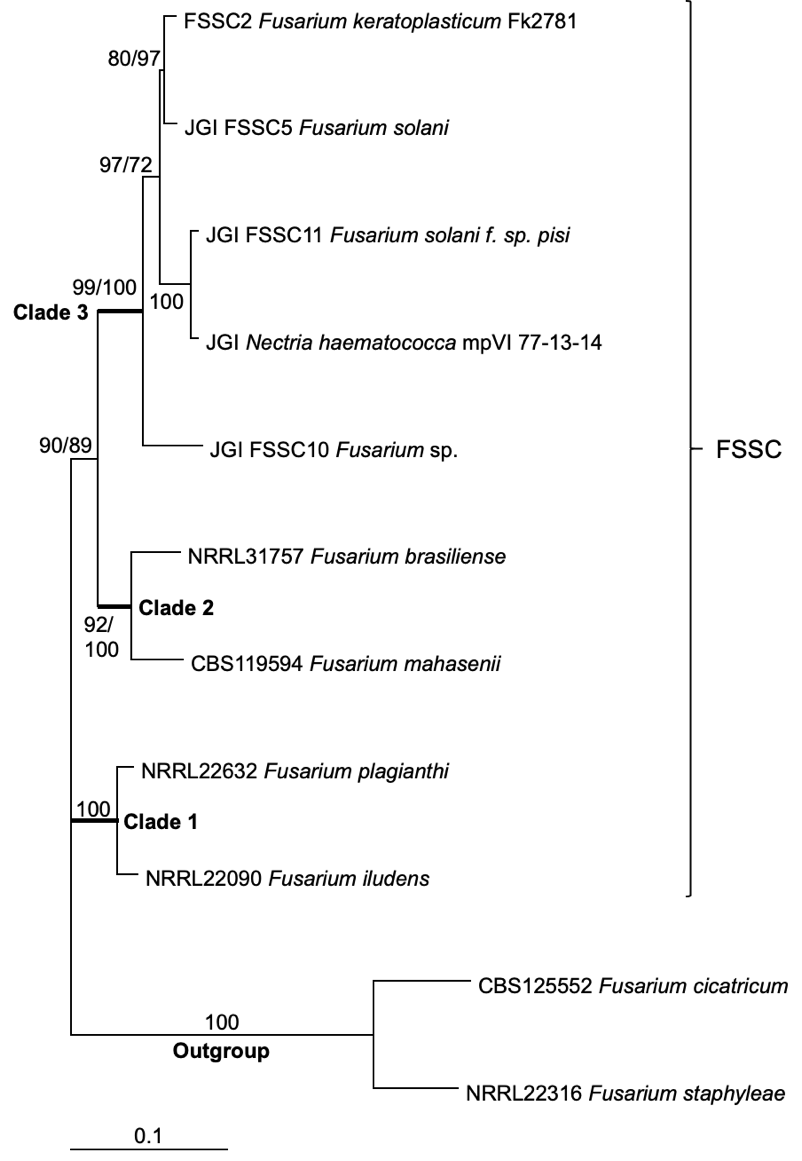


**Supplementary Figure S2.** A phylogram of *Fusarium* species representatives inferred from a Maximum Likelihood analysis of concatenated *TEF1-α* and *RPB2* sequences. The 2390 bp concatenated sequences include sequences of 14 separate coding regions. The branches of the three major FSSC clades are in bold. The percentages of branch support were calculated from 1,000 bootstrap (BS) replicates using both the MP and ML bootstrap analyses (MPBS/MLBS); only one value is stated if MPBS = MLBS. Scale bar indicates the number of nucleotide substitutions per position.
